# Cystic Clival Chordoma Presenting With Cerebrospinal Fluid Rhinorrhea: A Case Report and Literature Review

**DOI:** 10.7759/cureus.105956

**Published:** 2026-03-27

**Authors:** Yoshiki Mochizuki, Reina Mizuno, Mitsuaki Shirahata, Taku Homma, Tomonari Suzuki, Kazuhiko Mishima

**Affiliations:** 1 Neuro-Oncology/Neurosurgery, Saitama Medical University International Medical Center, Hidaka, JPN; 2 Pathology, Saitama Medical University International Medical Center, Hidaka, JPN; 3 Pediatric Neuro-Oncology/Neurosurgery, Saitama Medical University International Medical Center, Hidaka, JPN

**Keywords:** bone invasion, clival chordoma, ecchordosis physaliphora, gross total resection (gtr), rhinorrhea

## Abstract

Chordomas are rare malignant tumors that typically arise at the skull base and rarely present with cerebrospinal fluid (CSF) rhinorrhea. We report the case of a 40-year-old man with a clival chordoma who initially presented with headaches and serous rhinorrhea. Imaging revealed a non-enhancing cystic lesion extending from the prepontine cistern to the sphenoid sinus. Endoscopic endonasal removal of the cystic lesion and dural reconstruction was performed. Histopathology of the lesion confirmed a conventional chordoma, with positive brachyury and preserved nuclear SMARCB1 staining. The diagnosis was based on the surgical findings and histological analysis, including the Ki-67 labeling index and bone invasion. The patient recovered uneventfully and has remained free of both tumor recurrence and CSF rhinorrhea for more than two years.

This case highlights the importance of including chordomas in the differential diagnosis of CSF rhinorrhea due to clival cystic lesions. It also emphasizes the critical role of histopathological examination in distinguishing chordomas from other clival lesions, such as ecchordosis physaliphora (EP). Gross total resection (GTR) should be performed when feasible, and histologic evidence of bone invasion can be an important diagnostic clue for chordomas.

## Introduction

Chordomas are rare, slow-growing, malignant tumors that arise from embryonic notochordal remnants and account for approximately 0.5% of all primary intracranial tumors [1]. Intracranial chordomas frequently present with headache and cranial nerve palsies, particularly involving the abducens nerve, as a result of mass effects and local invasion [2]. Given their growth pattern as malignant tumors, accurate differentiation from benign lesions is essential.

In contrast, cerebrospinal fluid (CSF) rhinorrhea is an exceedingly rare initial manifestation of skull base chordoma [3-10]. Chordomas typically demonstrate solid components with contrast enhancement; however, in cases with atypical imaging features, such as a cystic appearance and lack of contrast enhancement, differentiation from benign notochordal lesions, such as ecchordosis physaliphora (EP), can be challenging [3,4,10].

In this context, histopathological evidence of bone invasion may serve as an important diagnostic clue favoring chordoma. In this report, we describe a case of cystic clival chordoma presenting with CSF rhinorrhea as the initial symptom and discuss the diagnostic challenges and importance of gross total resection (GTR).

## Case presentation

**Figure 1:**
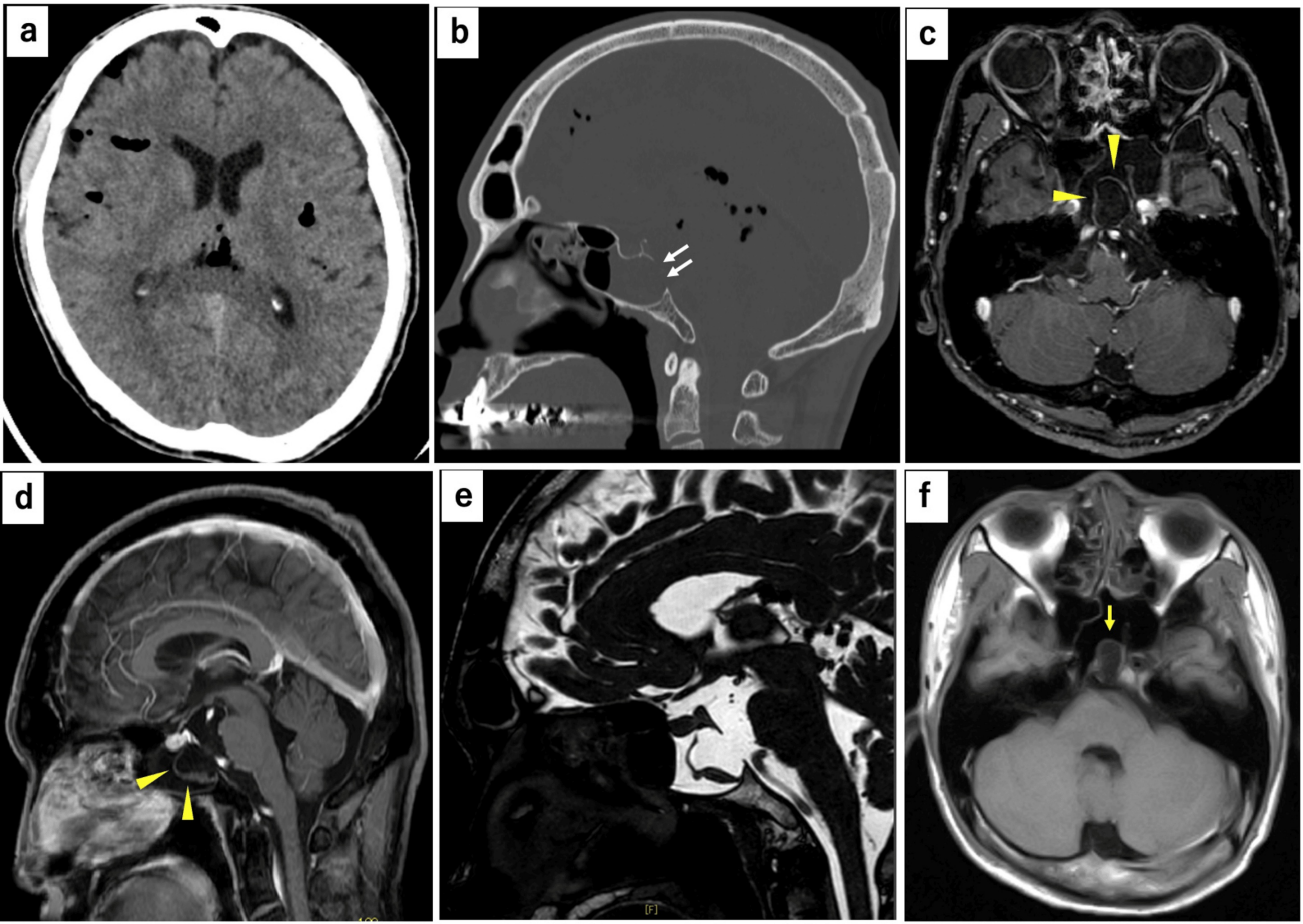
Preoperative CT and MRI findings of clival cystic lesion with cerebrospinal fluid rhinorrhea (a)-(b) CT on admission shows pneumocephalus, a defect of the posterior wall of the clivus (white arrows), and fluid accumulation in the sphenoid sinus. (c)-(e) MRI shows a cyst-like lesion protruding from the prepontine cistern to the sphenoid sinus, not enhanced by gadolinium (yellow arrowheads). (f) MRI at 33 years of age, revealing a cystic lesion in the sphenoid sinus (yellow arrow). CT: computed tomography; MRI: magnetic resonance imaging

A 40-year-old man presented with a three-week history of serous rhinorrhea and headache. Physical examination revealed no neurological deficits. Computed tomography (CT) revealed pneumocephalus, a focal defect in the clivus, and fluid accumulation in the sphenoid sinus (Figures 1a-1b). Magnetic resonance imaging (MRI) showed a cystic lesion extending from the prepontine cistern to the sphenoid sinus. The cyst wall showed no gadolinium enhancement, and the cyst was characterized as T1-hypointense, T2-hyperintense, and unilocular (Figures 1c-1e). The patient had previously undergone an MRI for headaches at ages 33 and 36 years, which demonstrated a small cystic structure in the sphenoid sinus, protruding through a clival bony defect, although this finding was not recognized at that time (Figure 1f).

**Figure 2:**
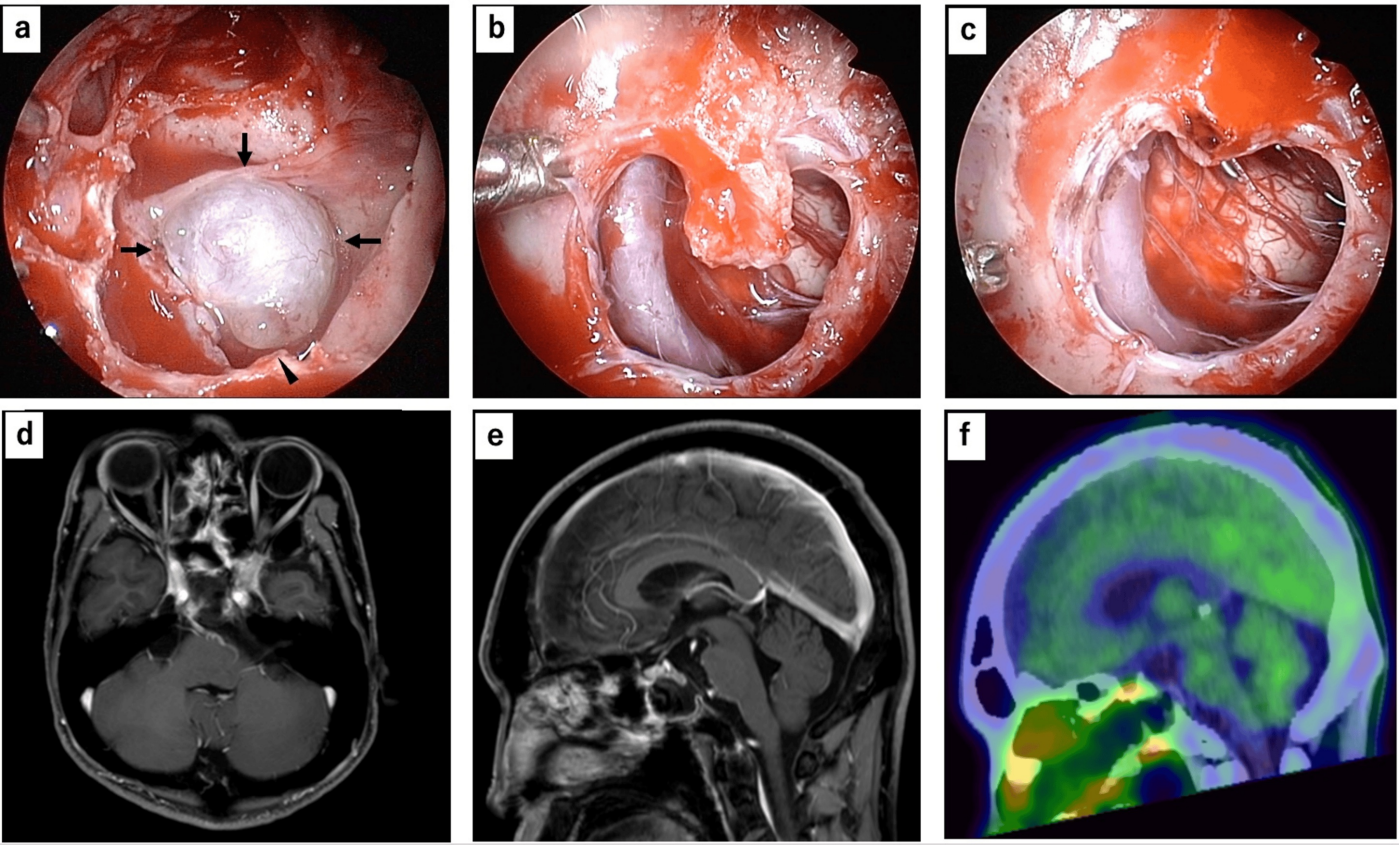
Intraoperative findings and postoperative imaging (a) The cyst wall protrudes into the sphenoid sinus through the clivus (black arrows). Cerebrospinal fluid leakage was observed from the inferior wall of the cyst (black arrowhead). (b) The cyst wall is removed and the prepontine cistern is observed through the defective clivus. (c) Gross total removal of the tumor is achieved and the thinned clivus is trimmed. (d, e) Postoperative magnetic resonance image showing no residual lesion. (f) 11C-methionine positron emission tomography computed tomography showing no methionine uptake around the clivus.

The rhinorrhea persisted despite conservative management at the referring hospital, and the patient was subsequently transferred to our institution, where we performed semi-urgent endoscopic endonasal surgery via the sphenoid sinus. After removal of the anterior sphenoid wall, the clivus appeared markedly thinned, with a focal defect. The portion of the cyst wall protruded into the sphenoid sinus through the region of the clival defect, and a substantial grayish-white component was observed around the cyst wall (Figure 2a). The dura mater was largely preserved, although focal dural and bony invasion was observed. The cyst wall, including dura and the area of bony invasion, was resected, and the CSF leak was subsequently reconstructed using fascia lata. Intraoperative pathological examination revealed large, vacuolated cells resembling macrophages, which raised the suspicion of a neoplastic lesion, such as a chordoma. GTR of the cystic mass lesion was performed, and the thinned clivus was trimmed as much as possible (Figures 2b-2c). The clival defect was repaired with the septal cartilage, fat tissue, an artificial dural substitute, and a left nasoseptal mucous flap, followed by fibrin glue sealant.

Postoperative MRI confirmed GTR of the tumor, including the component within the sphenoid sinus (Figures 2d-2e). 11C-methionine positron emission tomography/computed tomography (Met-PET/CT) showed no uptake around the clivus (Figure 2f).

**Figure 3:**
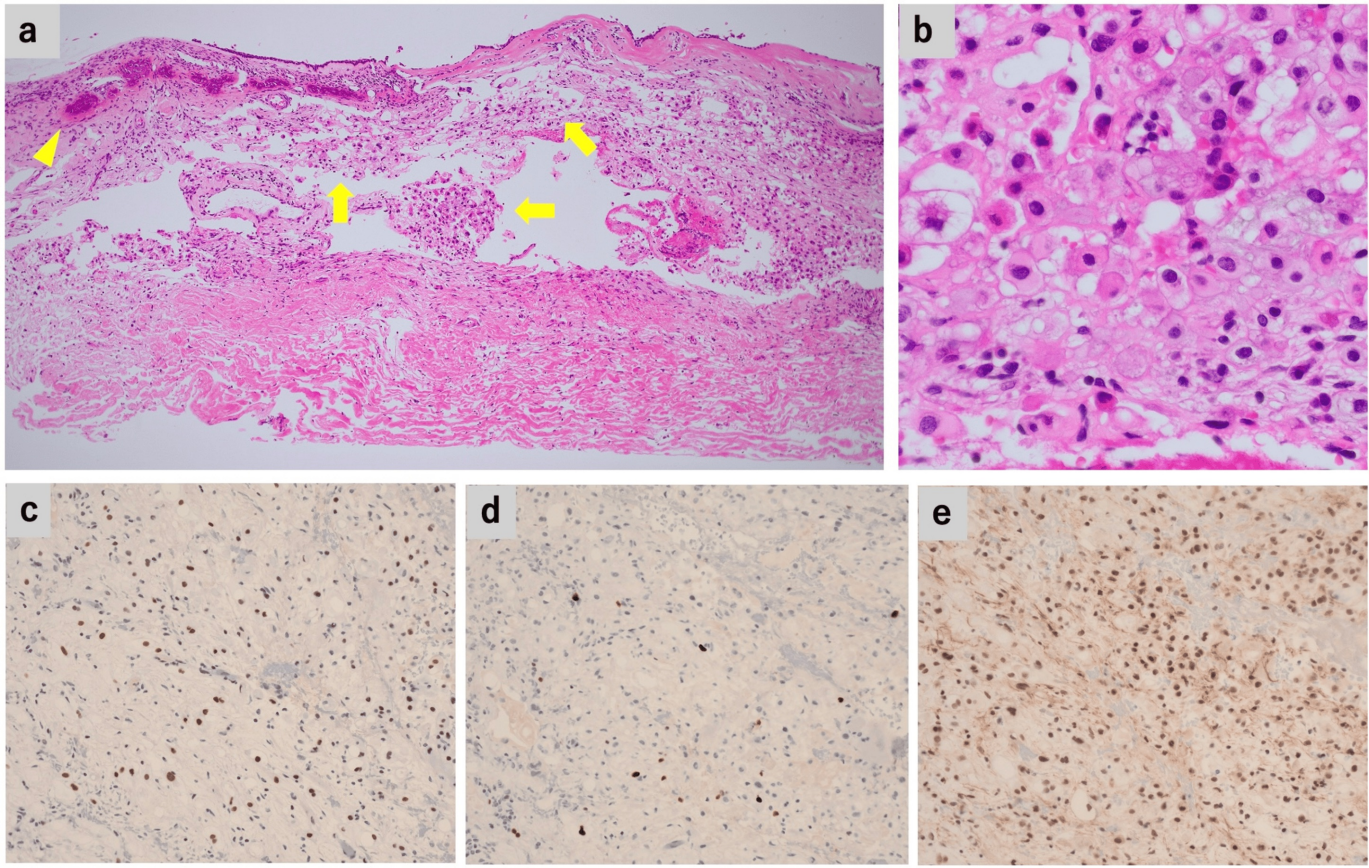
Histopathological and immunohistochemical findings of the tumor (a) The tumor cell (yellow arrows) is observed originating within the bone (yellow arrowhead), expanding with associated bone destruction, and proliferating between the mucosal layer and the dura mater (H&E, 10×). (b) Nests and cords of lightly eosinophilic chordoma cells are evident, with scattered physaliphorous cells (H&E, 60×). (c) The tumor cells are positive for brachyury (20×). (d) The Ki-67 labeling index is 5%-7% (20×). (e) Nuclear staining of SMARCB1 is retained (20×). H&E: hematoxylin and eosin

Hematoxylin and eosin staining revealed sheet-like arrangements of neoplastic cells with round nuclei and lightly eosinophilic cytoplasm between the defective bone and the dura mater (Figures 3a-3b). Bone invasion was also identified, confirming the tumor’s infiltrative nature (Figure 3a). The tumor cells exhibited varying degrees of intracytoplasmic vacuolization, consistent with physaliphorous cell morphology. Immunohistochemical analysis revealed that the tumor cells stained positively for brachyury (Figure 3c), S100 protein, and AE1/AE3, with a Ki-67 labeling index (LI) of 5%-7% (Figure 3d). Nuclear staining for SMARCB1 was preserved (Figure 3e). These findings were compatible with a classification of conventional chordoma in the fifth edition of the WHO Classification of CNS Tumours.

Although adjuvant therapies, such as radiation, heavy particle beams, or proton beams, were considered, they were not performed because GTR had been achieved. A small amount of rhinorrhea recurred 11 days after surgery, and a spinal drain was placed. The symptoms resolved, and the patient was discharged 36 days after admission. The patient remained free of rhinorrhea and recurrence during the two-year follow-up period, with regular MRI.

## Discussion

In this report, we present a rare case of clival chordoma presenting with CSF leakage as the initial manifestation. Chordomas should be considered a potential cause of clivus defects and CSF rhinorrhea, even in the absence of a mass region with contrast enhancement on MRI. This case report demonstrates the importance of achieving GTR and evaluating bone invasion pathologically, even in non-enhancing cystic lesions on MRI.

Chordoma is a rare neoplasm that accounts for approximately 0.5% of primary intracranial tumors [1]. It arises from embryonic notochordal remnants and typically occurs at the skull base and sacrococcygeal region. The most common symptoms of intracranial chordomas are neuro-ophthalmological deficits (62%) and headache (17.5%), which are especially prevalent in clival chordomas due to their proximity to the cranial nerves and brainstem [2]. Among the cranial nerves, the abducens nerve is the most frequently affected, demonstrating involvement in up to approximately 40% of cases at the initial presentation [2].

By contrast, CSF leakage is an exceedingly rare initial symptom. To the best of our knowledge, only eight cases presenting with CSF leakage as the initial symptom have been reported (Table 1) [3-10]. The patients were between 40 and 60 years of age. Among the eight reported cases, five (63%) showed no gadolinium enhancement on MRI. The Ki-67 LI also ranged widely, from as low as 0.4% to as high as approximately 5%, and several tumors exhibited cystic features, irrespective of contrast enhancement. As shown in Table 1, the variability of imaging features and Ki-67 LI indices likely reflects the heterogeneity of chordomas, suggesting that a diagnosis cannot be made based on any single finding alone. Furthermore, in our case, the size of the cyst had remained unchanged over a seven-year period. These imaging findings raised the possibility of a benign cystic lesion in the sphenoid sinus, illustrating a potential diagnostic pitfall.

**Table 1:** Cases of clival chordoma presenting with cerebrospinal fluid rhinorrhea F: female; M: male; MRI: magnetic resonance imaging; IHC: immunohistochemistry; Gd: gadolinium; TSS: transsphenoidal surgery; N/A: not applicable; EMA: epithelial membrane antigen

Author/Year	Age/Sex	Initial Symptom	MRI	IHC	Ki-67 (%)	Treatment	Outcome
Hsieh et al. (2009) [6]	47/F	Rhinorrhea, headache	Cystic lesion, Gd +	N/A	N/A	TSS resection and repair	Improved symptom
Feng et al. (2010) [8]	62/M	Rhinorrhea	No apparent mass or lesion enhancement	EMA+, S100+, vimentin+	5	TSS resection and repair	Disease-free (6 months)
Alshammari et al. (2012) [5]	60/F	Rhinorrhea, cough, and unconsciousness	An osteolytic, irregular lesion, Gd -	N/A	N/A	TSS resection and repair	Disease-free (3 months)
Ando et al. (2019) [7]	52/M	Headache, impaired consciousness	No apparent mass or lesion enhancement	Brachyury+	N/A	TSS resection and repair	Disease-free (12 months)
Andijani et al. (2020) [9]	58/M	Tonic-clonic seizure	No apparent mass lesion	Brachyury+, pancytokeratin+	N/A	TSS resection and repair	Referred for radiotherapy
Ujikawa et al. (2020) [3]	41/M	Rhinorrhea, headache	Cystic lesion, Gd +	Brachyury+, AE1/AE3+, S100+	0.4	TSS resection and repair	Disease-free (36 months)
Prather et al. (2023) [4]	43/F	Rhinorrhea, dysgeusia	No enhancing lesion	Brachyury+, pancytokeratin+	4.3	TSS resection, repair, and adjunctive proton beam radiation	Disease-free (24 months)
Abtahi et al. (2024) [10]	62/M	Postural headache, rhinorrhea, photophobia	Cystic lesion, Gd +	Brachyury+, EMA, S100+, SMARCB1 retained	Low	TSS resection and repair	Referred for radiotherapy
Present case	40/M	Rhinorrhea, headache	Cystic no-enhancing lesion with a membrane	Brachyury+, AE1/AE3+, S100+ SMARCB1 retained	5~7	TSS resection and repair	Disease-free (24 months)

Considering these characteristics, in cases of chordoma presenting with CSF leakage, the clinical course may differ from that of typical skull base chordoma. Of the eight reported cases, the differential diagnosis between chordoma and EP was specifically discussed in three cases [3,4,10].

EP is a benign congenital lesion derived from ectopic notochordal remnants, most commonly located in the spheno-occipital synchondrosis of the clivus. While EP is usually asymptomatic, Castello Ruiz et al. reported that CSF rhinorrhea is an initial presentation in 35% of cases of symptomatic EP [11]. Recently, the differentiation between chordoma and EP has provoked much discussion [3,4,10,12-15]. As demonstrated in reported cases of chordoma presenting with CSF leakage, these lesions are difficult to differentiate clinically and radiologically (Table 1). Lagman et al. reported a lower Ki-67 LI in EP (typically < 1%) and higher in chordoma, suggesting that the Ki-67 LI is useful for differentiating chordoma from EP [15]. However, as shown in Table 1, chordomas with a Ki-67 LI of < 1% have also been reported, indicating that this marker alone is insufficient for a reliable diagnosis [3]. Although a Ki-67 LI of less than 1% is considered a necessary criterion for the diagnosis of EP, this finding alone is not adequate to exclude chordoma [15].

Previous reports have noted that a characteristic feature of EP, in contrast to chordoma, is the absence of bone invasion [13]. Accordingly, demonstrating the presence or absence of bone invasion on histopathological examination may provide an important clue in differentiating chordoma from EP. A focal bony defect adjacent to EP may occasionally be observed, which could predispose the patient to meningitis or CSF leakage, even following minor head trauma [16]. In our case, histopathological bone invasion by the neoplastic cells was confirmed, and, together with the elevated Ki-67 LI, the diagnosis of chordoma was supported.

Although GTR is the most consistently recognized prognostic factor for chordomas, other markers have been proposed. A high Ki-67 LI (>5%) is associated with shorter progression-free survival (PFS) [17], and fluorescence in situ hybridization for 1p36 and 9p21 deletions may stratify patients into prognostic groups [18]. Recent studies have also identified molecular alterations, such as PBRM1 and PIK3CA mutations, which may serve as potential prognostic and therapeutic markers [19].

Methionine PET-CT is a useful imaging modality for chordomas [20]. In our case, Met-PET-CT revealed no residual uptake in the clivus, supporting the achievement of GTR. Furthermore, the lesion in our patient exhibited extremely slow growth over a prolonged period, suggesting a relatively indolent clinical behavior. Notably, no recurrence has been observed during more than two years of follow-up.

## Conclusions

In patients presenting with cystic lesions within the sphenoid sinus accompanied by clival defects, it is important to consider chordoma in the differential diagnosis, even in the absence of typical radiological features. Given the potential diagnostic overlap with benign notochordal lesions such as EP, no individual finding is sufficient for a definitive diagnosis; therefore, integrated assessment of clinical and pathological findings is essential. GTR should be pursued whenever feasible, as it not only provides therapeutic benefit but also enables definitive histopathological diagnosis. Moreover, removal of the affected clival bone may be particularly useful in demonstrating tumor cell invasion, which can serve as a key diagnostic clue in distinguishing chordoma from EP. This case underscores the importance of a comprehensive surgical and pathological approach in the management of atypical clival lesions.

## References

[1] Khawaja AM, Venkatraman A, Mirza M (2017). Clival chordoma: case report and review of recent developments in surgical and adjuvant treatments. Pol J Radiol.

[2] Colli BO, Al-Mefty O (2001). Chordomas of the skull base: follow-up review and prognostic factors. Neurosurg Focus.

[3] Ujikawa T, Tanaka Y, Onaka K, Kudo T, Sugawara T, Sumita K, Maehara T (2020). A case report of clivus chordoma presenting with cerebrospinal fluid rhinorrhea (Article in Japanese). No Shinkei Geka.

[4] Prather KY, Shi HH, McKinney KA, Dunn IF (2023). Chronic cerebrospinal fluid rhinorrhea as an initial presentation of chordoma: illustrative case. J Neurosurg Case Lessons.

[5] Alshammari J, Monnier P, Daniel RT, Sandu K (2012). Clival chordoma with an atypical presentation: a case report. J Med Case Rep.

[6] Hsieh CT, Liu MY, Su WF, Ju DT (2009). Spontaneous cerebrospinal fluid rhinorrhea: a rare initial presentation of clival chordoma. Neurol India.

[7] Ando S, Usuda H, Umeda Y, Umeda M, Oyake M, Fujita N (2019). A case of chordoma presenting as recurrent bacterial meningitis with cerebrospinal fluid leakage (Article in Japanese). Rinsho Shinkeigaku.

[8] Feng K, Qiuhang Z, Qiuyi Q (2010). Transclival cerebrospinal fluid rhinorrhea as the initial presenting symptom of a tiny intradural chordoma. J Clin Neurosci.

[9] Andijani M, Jamjoom A, Togersen A, Ram B, Bodkin P, Kamel M (2020). An unusual presentation of clival chordoma: a case report and review of the literature. Br J Neurosurg.

[10] Abtahi SS, Ragulojan M, Alghamdi A, Woulfe J, Alnemari A, Alkherayf F (2024). Differential diagnosis of a cystic notochordal skull base lesion: a case report. Can J Neurol Sci.

[11] Castello Ruiz MJ, Alsavaf MB, Fadel M (2023). Spontaneous rhinorrhea: a possible concealing initial symptom of ecchordosis physaliphora. J Neurosurg Case Lessons.

[12] Kreshak J, Larousserie F, Picci P (2014). Difficulty distinguishing benign notochordal cell tumor from chordoma further suggests a link between them. Cancer Imaging.

[13] Stevens AR, Branstetter BF 4th, Gardner P, Pearce TM, Zenonos GA, Arani K (2023). Ecchordosis physaliphora: does it even exist?. AJNR Am J Neuroradiol.

[14] Stuebe CM, Rindler RS, Laack N, Carr CM, Choby G, Inwards CY, Van Gompel JJ (2023). Evaluation of long-term follow-up in ecchordosis physaliphora versus chordoma. World Neurosurg.

[15] Lagman C, Varshneya K, Sarmiento JM, Turtz AR, Chitale RV (2016). Proposed diagnostic criteria, classification schema, and review of literature of notochord-derived ecchordosis physaliphora. Cureus.

[16] Yuh SJ, Woulfe J, Corsten MJ, Carrau RL, Prevedello DM, Kassam AB (2014). Diagnostic imaging dilemma of a clival lesion and its clinical management implications. J Neurol Surg B Skull Base.

[17] Horbinski C, Oakley GJ, Cieply K, Mantha GS, Nikiforova MN, Dacic S, Seethala RR (2010). The prognostic value of Ki-67, p53, epidermal growth factor receptor, 1p36, 9p21, 10q23, and 17p13 in skull base chordomas. Arch Pathol Lab Med.

[18] Zenonos GA, Fernandez-Miranda JC, Mukherjee D (2019). Prospective validation of a molecular prognostication panel for clival chordoma. J Neurosurg.

[19] Passeri T, Gutman T, Hamza A (2023). The mutational landscape of skull base and spinal chordomas and the identification of potential prognostic and theranostic biomarkers. J Neurosurg.

[20] Zhang H, Yoshikawa K, Tamura K (2004). Carbon-11-methionine positron emission tomography imaging of chordoma. Skeletal Radiol.

